# A biomechanical study comparing two fixation methods in depression fractures of the lateral tibial plateau in porcine bone

**DOI:** 10.1186/2052-1847-5-15

**Published:** 2013-08-30

**Authors:** Caroline M Blakey, Michael Rennison, Stephen P Guy, Paul M Sutton

**Affiliations:** 1 Department of Orthopaedics, Sheffield Teaching Hospital NHS Trust, Herries Road, Sheffield S5 7AU, UK; 2 Department of Mechanical Engineering, University of Sheffield, Sheffield S1 3JD, UK; 3 Department of Orthopaedics, Bradford Teaching Hospitals NHS Trust, Duckworth Lane, Bradford BD9 6RJ, UK

## Abstract

**Background:**

A novel method of fixation has been described for the treatment of pure depression fractures of the lateral tibial plateau. Fracture fragments are elevated through a reamed transtibial tunnel. An interference screw is then passed into the tunnel to buttress fracture fragments from beneath. This method of fixation has perceived benefits but there have been no studies to demonstrate that the technique is biomechanically sound. The aim of our study is to compare traditional parallel, subchondral screw fixation with the use of an interference screw, assessing maintenance of fracture reduction following simulated post-operative loading, and overall construct strength.

**Methods:**

Depression fractures of the lateral tibial plateau were simulated in 14 porcine knees. Fracture fragments were elevated through a reamed transtibial tunnel and samples were randomly assigned to a fixation method. 7 knees underwent traditional fixation with parallel subcortical cannulated screws, the remainder were stabilized using a single interference screw passed through the transtibial tunnel. Following preloading, each tibia was cyclically loaded from 0 to 500 Newtons for 5,000 cycles using a Nene testing machine. Displacement of the depressed fracture fragments were measured pre and post loading. Samples were then loaded to failure to test ultimate strength of each construct.

**Results:**

The depression displacement of the fractures fixed using cannulated screws was on average 0.76 mm, in comparison to 0.61mm in the interference screw group (p=0.514). Mechanical failure of the cannulated screw constructs occurred at a mean of 3400 N. Failure of the transtibial interference screw constructs occurred at a mean of 1700 N (p<0.01). In both groups the mechanism of ultimate failure was splitting of the tibial plateau.

**Conclusion:**

These results demonstrate the increased biomechanical strength of parallel, cannulated screws for depression fractures of the tibial plateau, however the use of a transtibial interference screw may be a viable method of fixation under physiological loads.

## Background

Arthroscopy is increasingly used as an adjunct to the treatment of tibial plateau fractures. Arthroscopy allows direct visualization of the articular surface with out need for arthrotomy or meniscal detachment. It allows evacuation of haemarthrosis or fracture debris from joint, and treatment of associated meniscal pathologies [1,2].

Arthroscopic reduction, internal fixation is particularly useful for Schatzker III fractures where there is less risk of fluid extravasation and compartment syndrome than fractures that split the cortex. Fracture fragments can be elevated through a reamed transtibial tunnel under direct arthroscopic visualization [3]. Percutaneous, cannulated screws can then be passed under the subchondral plate, to buttress fracture fragments, in the method traditionally described [4].

More recently a newer method of fixation has been described which involves the use of an interference screw placed beneath elevated bone fragments in place of traditional transverse subchondral screws [5]. Use of an interference screw allows simultaneous, precise fracture reduction under direct arthroscopic visualization and reamed metaphyseal bone can be used as autograft. The technique eliminates the need for percutaneous buttress screw placement under fluoroscopic guidance, and avoids the soft tissue irritation that is sometimes associated with prominent percutaneous screws.

We are unaware of any published studies that demonstrate this technique is biomechanically sound. We compared fractures fixed with traditional parallel, subchondral screws, with those fixed with a single transtibial interference screw. We aimed to measure depression in the articular surface, following simulated post-operative loading. Secondly we aimed to compare the overall biomechanical strength between these two methods of fracture fixation. We hypothesised that there would be no significant difference between these two constructs.

## Methods

### Specimen Preparation

**Figure 1:**
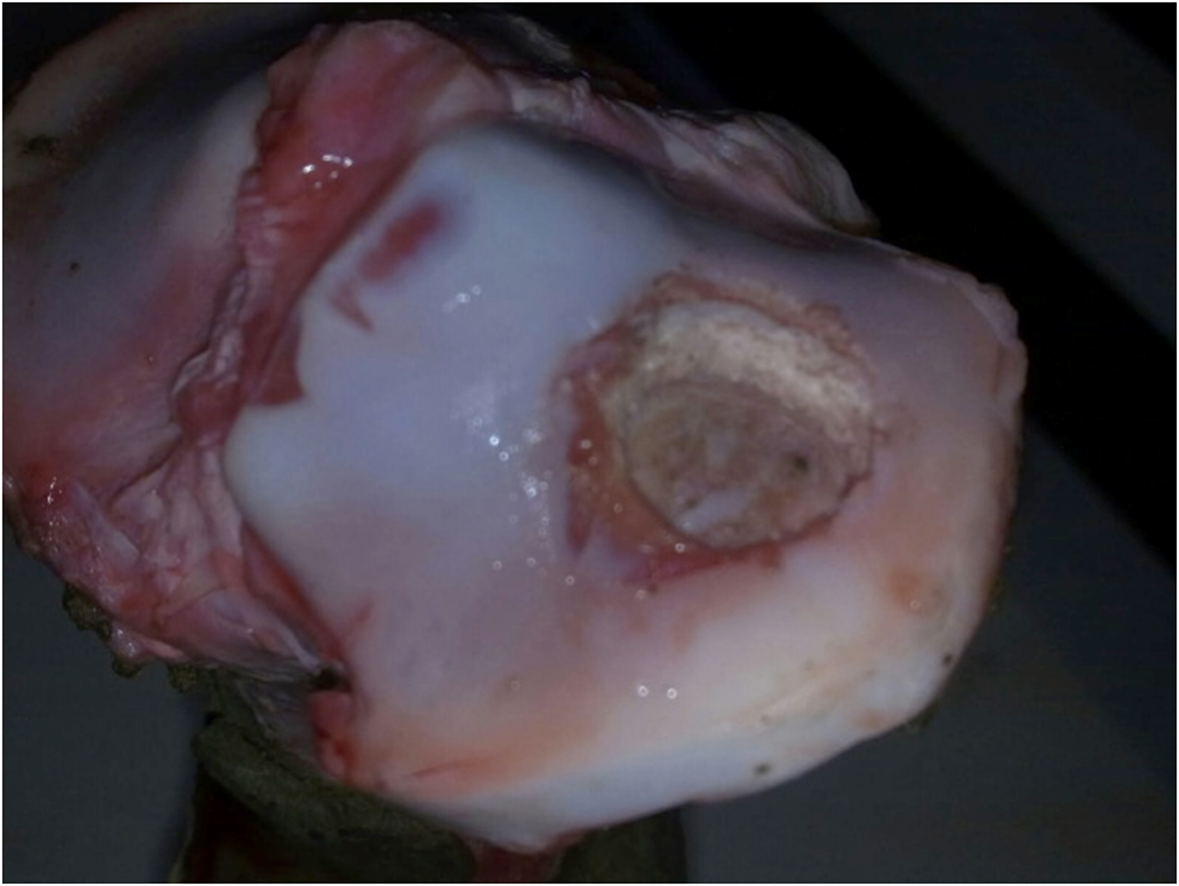
Digital photograph showing the simulated fracture through the lateral tibial plateau.

14 porcine knees were prepared for study. Knees were disarticulated and all soft tissues were removed. Specimens were stored at −18°C and thawed for 24 hours before testing. Depression fractures of the lateral tibial plateau were simulated using a hydraulic compression testing machine (Nene testing systems®). Tibiae were mounted in the machine using a custom made jig. Increasing load was applied through the lateral tibial plateau with an 18 mm ram. Force feedback monitored through the digital controller identified the point of breach of the articular surface. This method was found to reproducibly result in an isolated depression fracture of the tibial plateau, without cortical split. (Figure 1) Depth of depression was on average of 2 mm.

In each specimen, fracture fragments were elevated through a reamed transtibial tunnel. An anterior cruciate ligament (ACL) jig was used to pass a guide pin through the anterolateral tibial metaphysis into the center of the depressed fragment. A 5 mm coring reamer was passed over the guide wire to penetrate the tibial cortex, through metaphyseal bone, to within 5 mm of the articular surface. An angled punch was used to reduce depressed fragments from below.

### Study groups

Specimens were randomly assigned to fixation method. 7 knees underwent traditional fixation with two 4.5 mm subchondral, cannulated screws, centered 5 mm below the articular surface. (Stryker 4.5 mm stainless steel 4.5 mm screws to reflect the proportional decrease in size between porcine and human knees assuming traditional fixation with 6.5 mm screws. The remaining 7 knees were stabilized using a single titanium interference screw, passed through the transtibial tunnel and oversized 1 mm.

### Biomechanical testing

**Figure 2:**
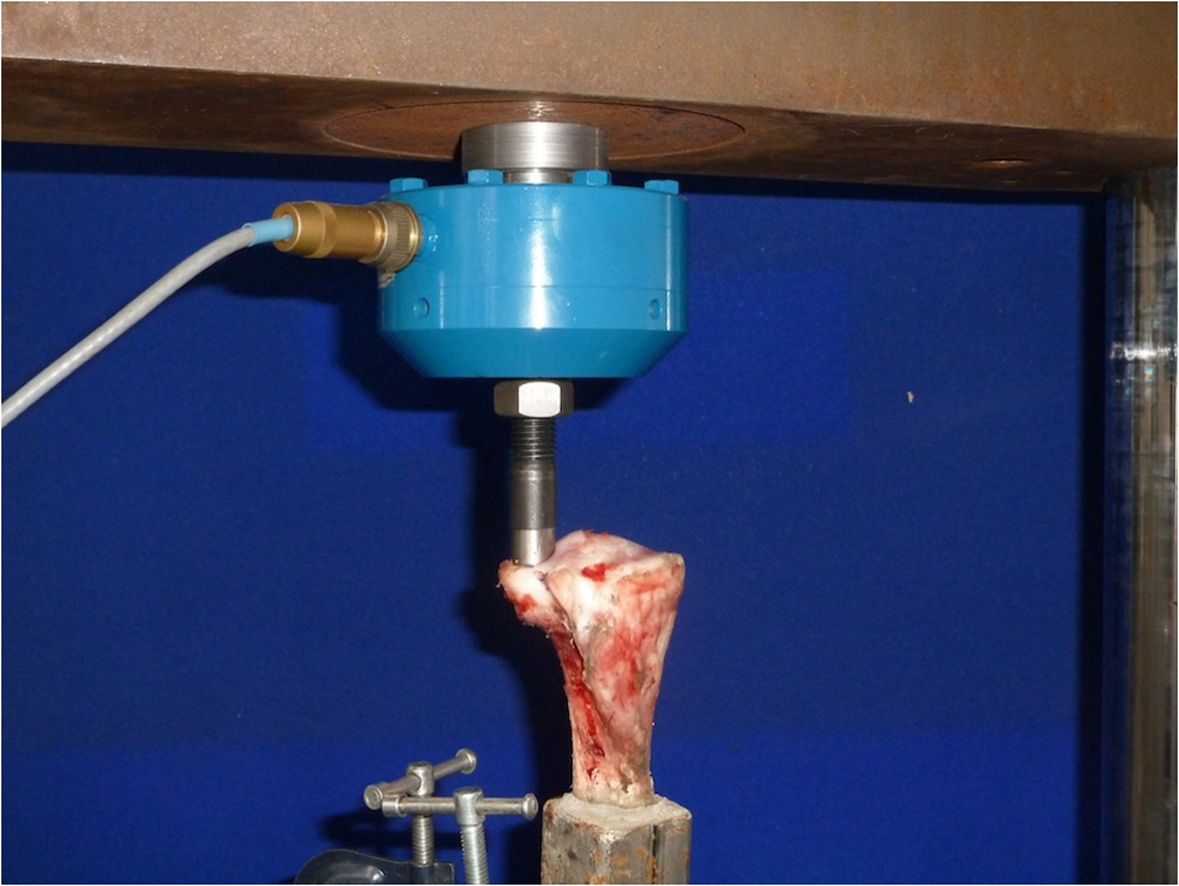
Digital photograph showing a specimen mounted on hydraulic compression testing machine.

Following fixation, specimens were remounted onto the hydraulic testing machine. (Figure 2) Specimens were preloaded from 0 to 500 N for 50 cycles and any depression in the articular surface was measured with a dial test indicator accurate to within 0.01 mm (Kennedy dial®). Each specimen then underwent cyclical loading, from 0 to 500 N for 5,000 cycles at 3 Hz, before the articular depression was re-measured. The difference in articular depression measured pre and post loading was calculated. Finally, samples were loaded to failure, applying increasing static load through the fracture. Load–displacement data were recorded continuously and stored every 15 milliseconds.

### Statistical analysis

A sample size calculation was made based on a clinically significant articular depression of 2 mm. The Mann–Whitney U test was used for statistical analysis of results. A minimum significance level of p = 0.05 was set for all statistical tests. Statistics were calculated using Excel for Windows.

## Results

### Cyclical loading

There was no statistically significant difference between mean displacement of the articular depression, in fractures fixed using subchondral screws, and those in the interference screw group. (Table 1) No specimens failed during cyclical loading.

**Table 1:** Results

	**Interference screws**	**Parallel subchondral screws**	**p value**
**Displacement (range)**	0.61 mm	0.76 mm	0.62
	(0.35 to 1.4 mm)	(0.02 to 1.2 mm)	
**Ultimate load to failure (N)**	1700 N	3400 N	<0.01
	(1000 to 2143 N)	(2953 to 4428 N)	

### Load to failure

**Figure 3:**
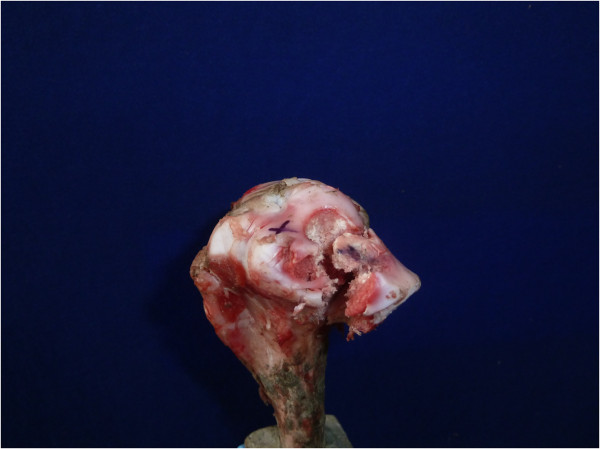
Digital photograph demonstrating construct failure mechanism.

Overall construct strength of the group fixed with parallel screws was on average twice that of in the interference screw group (Table 1). In both groups the mechanism of ultimate failure was splitting of the tibial plateau (Figure 3).

## Discussion

The purpose of this study was to evaluate the biomechanical strength of the use of a transtibial interference screw, for fixation of depression fractures of the lateral tibial plateau. Methods used were similar to previous published studies on tibial plateau fracture fixation [6,7]. Our results demonstrate this novel fixation method provides comparable biomechanical results to traditional, parallel, subchondral screws, under normal physiological loads.

The technique of using a transtibial interference screw for fracture fixation of pure depression fractures of the tibial plateau was described in 2006 [5], but no clinical or biomechanical data has since been published to support its use. Previous studies have compared the number and size of subchondral screws used in traditional fixation, and the benefits of the use of bone graft, cement or trabecular metal to support elevated fragments [6-8]. The newer method of fixation, has perceived benefits. Arthroscopic reduction preserves the soft tissue envelope, negating the need for arthrotomy and meniscal detachment. Use of an interference screw allows simultaneous, precise fracture reduction under direct arthroscopic visualization. Reamed metaphyseal bone can be used as autograft. The technique eliminates the need for percutaneous buttress screw placement under fluoroscopic guidance, and avoids the soft tissue irritation that is sometimes associated with prominent percutaneous screws. The technique allows the possible introduction of bioabsorbable screw use. In this study only titanium screws were tested.

Our results demonstrated articular depression, following simulated post-operative loading, of on average 0.61 mm in fractures fixed with a single interference screw, and 0.76 mm in those fixed with parallel, subchondral screws. The goals of treatment of any intra-articular fracture include restoration of joint congruity, and prevention of secondary arthritis. Within the literature there is no definitive guidance on what is acceptable step off in articular cartilage following trauma. There are a number of studies, which have looked at this specific to the tibial plateau. Experimental models have shown acceptable depression of up to 3 mm before increased contact pressures are demonstrated in surrounding articular cartilage [9]. Clinical studies, comparing depression fractures managed operatively and conservatively, have shown deterioration in outcome with articular depression greater than 3 mm [10]. Others have shown no clinical difference despite up to 8 mm depression [11]. Following cyclical loading, the average depression of stabilized fracture fragments in both groups of our study, was comfortably within the acceptable range.

A load of 20 kg/cm^2^ has been shown to correspond to the maximal loading of the knee during normal gait in a 70 kg man [12]. Our study reflects the biomechanical strength of fracture constructs immediately after fixation and cyclical loading simulates early, full weight bearing in a patient of this size. No specimens failed during cyclical loading. Increased body weight or angular mal-alignment will increase the load passing through the lateral plateau. 15° of angular deformity increases maximal loading to 75 kg/cm^2^, [12] this would correspond to 1900 N in our study. Specimens ultimately failed at a mean of 1700 N in fractures fixed with a single interference screw. Protected weight bearing, or alternative fixation methods, should be considered in patients in whom load is expected to be out with normal physiological range.

We accept that our study has some limitations. Schatzker 3 fractures of the lateral tibial plateau, in which the tibial cortex remains intact with depression of the articular surface, are most commonly described in the elderly, with low level trauma [13]. The porcine knees used in this study may not be representative of the poor quality, osteoporotic bone seen in this group. Specimens in this study were disarticulated and stripped of soft tissue prior to simulation of the depression fracture. No cortical fracture could be seen on close inspection of the tibia but the possibility of microfracture could not be excluded without X-ray or CT. Using a cadaveric model has inherent drawbacks, tissue properties may have been affected by the preservation process. Mechanical testing has shown that fracture stress values in canine and porcine bone, most closely resemble those of human bone [14]. In both cadaveric and animal models there is often a wide variation in the mechanical properties of samples. In order to test mechanical construct strength, the ideal samples would be of identical bone quality. In this study, specimens were randomly assigned to fixation method in attempt to reduce the impact of variation in bone quality. The mechanism of fracture failure did not represent what we would expect in vivo. In this study we did not load the entirety of the tibial plateau, reducing the accuracy of simulated physiological loading. Loading over a larger area may prevent propagation of a cortical split from the original fracture depression. No consideration has been made to the effects of fracture healing.

## Conclusions

Our results demonstrate increased overall biomechanical strength of the use of traditional, parallel, subchondral screws, however the use of a transtibial interference screw may be a viable method of fixation under normal physiological loads.

## Competing interests

All authors declare that they have no competing interests.

## Authors’ contribution

PMS designed the study and supervised overall undertaking of the study and manuscript preparation. SPG assisted in specimen preparation and editing manuscript. CMB and MR set up mechanical studies and collected all data. CMB authored the main manuscript. All authors read and approved the final manuscript.

## Pre-publication history

The pre-publication history for this paper can be accessed here:

http://www.biomedcentral.com/2052-1847/5/15/prepub
